# Gestational Exercise Antagonises the Impact of Maternal High-Fat High-Sucrose Diet on Liver Mitochondrial Alterations and Quality Control Signalling in Male Offspring

**DOI:** 10.3390/ijerph20021388

**Published:** 2023-01-12

**Authors:** Jelena Stevanović-Silva, Jorge Beleza, Pedro Coxito, Paulo J. Oliveira, António Ascensão, José Magalhães

**Affiliations:** 1 Laboratory of Metabolism and Exercise (LaMetEx), Research Centre in Physical Activity, Health and Leisure (CIAFEL), Laboratory for Integrative and Translational Research in Population Health (ITR), Faculty of Sport, University of Porto, 4200-450 Porto, Portugal; 2 Department of Cell Biology, Physiology & Immunology, Faculty of Biology, University of Barcelona, 08028 Barcelona, Spain; 3 CNC—Center for Neuroscience and Cell Biology, CIBB—Centre for Innovative Biomedicine and Biotechnology, University of Coimbra, 3004-504 Coimbra, Portugal

**Keywords:** gestational diabetes, mitochondrial biogenesis, mitochondrial dynamics, epigenetics, gestational exercise, foetal programming

## Abstract

Maternal high-caloric nutrition and related gestational diabetes mellitus (GDM) are relevant modulators of the intrauterine environment, increasing the risk of liver metabolic alterations in mothers and offspring. In contrast, as a non-pharmacological approach against metabolic disorders, exercise is highly recommended in GDM treatment. We analysed whether gestational exercise (GE) protects mothers from diet-induced GDM metabolic consequences and mitigates liver mitochondrial deleterious alterations in their 6-week-old male offspring. Female Sprague Dawley rats were fed with control or high-fat high-sucrose (HFHS) diet and kept sedentary or submitted to GE. Male offspring were sedentary and fed with control diet. Sedentary HFHS mothers and their offspring showed impaired hepatic mitochondrial biogenesis and morphological evidence of mitochondrial remodelling. In contrast, GE-related beneficial effects were demonstrated by upregulation of mitochondrial biogenesis signalling markers and mitochondrial fusion proteins and downregulation of mitochondrial fission protein. Alterations in miR-34a, miR-130b, and miR-494, associated with epigenetic regulation of mitochondrial biogenesis, suggested that GE is a more critical modulator of intergenerational changes in miRs expression than the maternal diet. Our data showed that GE positively modulated the altered hepatic mitochondrial biogenesis and dynamics markers and quality control signalling associated with maternal HFHS-diet-related GDM in mothers and offspring.

## 1. Introduction

Maternal nutrition alterations during pregnancy result in a specific intrauterine milieu, which strongly impacts the offspring’s early life development, eventually increasing the risk of metabolic disorders in adulthood [1]. Exposure to maternal hyperglycaemia and insulin resistance (IR) in pregnancy, characteristic of gestational diabetes mellitus (GDM), represents a risk factor for the development of various metabolic disturbances in the offspring, including obesity, altered glucose metabolism, and non-alcoholic fatty liver disease (NAFLD) [2]. Given the essential role of mitochondria in metabolism and cellular quality control signalling, mitochondrial dysfunction may be one of the first events characterising the onset of metabolic diseases. Indeed, mitochondrial impairment has been observed in liver tissue under deleterious conditions of IR, diabetes, or NAFLD [3,4]. Importantly, although paternal mitochondrial DNA (mtDNA) can be transferred to the offspring in some exceptional cases, maternal inheritance remains more dominant [5]. This sheds light on the importance of maternal mitochondrial health in determining offspring mitochondrial fate, thereby influencing the phenotype of future generations.

However, data on GDM-related alterations in liver mitochondrial function of mothers and offspring are limited. Mitochondrial biogenesis, mitophagy, and dynamic processes modulate mitochondrial function to maintain mitochondrial homeostasis and plasticity to adapt to metabolic demands, which can be compromised in IR, diabetes, and liver diseases [3,4]. Despite similar pathophysiological mechanisms between GDM and non-gestational diabetes, the context of pregnancy brings a new aspect on the underlying mechanisms, which urges a need for research on GDM-related pathophysiology. Since GDM consequences are potentially transmitted across generations, their management becomes an important matter to be considered. Although some pharmacological approaches are recommended, changes in nutritional and physical exercise habits are considered essential first-line non-pharmacological approaches against GDM [6]. In fact, exercise has been recommended as a beneficial strategy against diabetes and liver diseases [3,7], as it can regulate the mitochondrial life cycle by enhancing mitochondrial biogenesis and dynamics, thus improving metabolic function [8]. Using a diet-induced GDM model, we previously reported that gestational exercise (GE) antagonised some of the impairments observed in mitochondrial function in GDM mothers and that these benefits were maintained for a prolonged period after exercise training cessation. Moreover, GE diminished the adverse effects of maternal diet on mitochondrial respiration and prevented lipid accumulation in male offspring liver [9]. However, the signalling processes, which are pivotal in determining the hepatic protective effects of exercise in NAFLD models, such as mitochondrial biogenesis and dynamics, (auto)mitophagy, and apoptotic signalling, are still to be elucidated in GDM mothers and their offspring. Therefore, here, we aimed to better understand the relevance of GE against GDM-induced deleterious consequences in both generations. We investigated the effects of maternal diet and GE on liver mitochondrial dynamics and biogenesis, as well as quality control signalling in male offspring and their eventual regulation through microRNA mechanisms. Additionally, we followed up on the effects of diet-induced GDM and GE on liver mitochondrial alterations in mothers 8 weeks after the pregnancy and exercise cessation.

## 2. Materials and Methods

### 2.1. Animal Model

Female 7-week-old Sprague Dawley rats were fed with either control (C) or high-fat high-sucrose (HFHS) diet (E157452-047 and D12451 (II) mod., Ssniff, Soest, Germany) for 7 weeks. Afterwards, following mating, pregnant (P) HFHS-fed dams were divided into sedentary animals (S) and those exercised during pregnancy (E). During pregnancy, as well as post-partum, dams continued consuming the same type of diet as before the pregnancy. This resulted in three experimental groups (n = 6): P-C-S: sedentary C-fed dams; P-HFHS-S: sedentary HFHS-fed dams; and P-HFHS-E: exercised HFHS-fed dams. P-HFHS-E dams were exercised on a motor-driven treadmill (LE8700, Panlab Harvard Apparatus, Holliston, MA, USA) 6 days/week, 40–60 min/day for 3 weeks, as previously described [9], and they had access to the free-running wheels during pregnancy. The sedentary groups were placed on a static treadmill to be exposed to the same environmental conditions. Dams delivered naturally, and the offspring were weaned after 3 weeks. Male pups were assigned to experimental groups depending on the maternal treatment (n = 6): C-S: offspring of C-fed sedentary dams; HFHS-S: offspring of HFHS-fed sedentary dams; and HFHS-E: offspring of HFHS-fed exercised mothers. The offspring were C-fed and sedentary. Dams were sacrificed at 8 weeks post-partum and offspring at 6 weeks of age.

All animals were housed in a normal environment (21–22 °C; 50–60% humidity; 12 h light/dark cycles), receiving food and water ad libitum. The Ethical Committee of the “Instituto de Investigação e Inovação em Saúde–i3S”, University of Porto, and National Government Authority (Direção Geral de Alimentação e Veterinánia; No. 0421/000/000/2018) approved the experimental protocol, in compliance with the Guidelines for Care and Use of Laboratory Animals in Research advised by the Federation of European Laboratory Animal Science Associations.

### 2.2. Animal Sacrifice and Tissue Sampling

After overnight fasting, anaesthesia was induced in animals by using 5% isoflurane and 1 L/m O_2_, and it was maintained by using 2.5% isoflurane and 0.4 L/m O_2_. Isolated liver tissue was rinsed with ice-cold PBS, and a median lobe was used for mitochondria isolation, as previously described [9]. The blood collected from inferior vena cava was centrifuged (3000× *g*, 10 min) to obtain blood plasma. Liver tissue and isolated mitochondria suspension, as well as collected plasma, were stored at −80 °C for later analysis.

### 2.3. Glycogen Content Determination

An amount of 30 mg of liver tissue was homogenised in 600 µL of ddH_2_O. The homogenate was boiled at 95 °C for 10 min and centrifuged at 15,500× *g*, 4 °C, for 10 min. The supernatant was diluted in PBS (1:6), and 30 µL of each sample was either digested in one volume of 1.2 U/mL amyloglucosidase (Sigma-Aldrich, A1602) or diluted in one volume of PBS. After setting up, the microplate (with blanks, glucose standards (0.01, 0.02, 0.04, 0.08, 0.16 mg/mL), and digested/diluted samples) was incubated first at 37 °C for 60 min and then at room temperature for 5 min. Afterwards, 100 µL of Glucose assay reagent (Sigma-Aldrich, St. Louis, MO, USA, G3293) was added to each blank, glucose standard, and sample, and the plate was incubated at room temperature for 15 min. The absorbance was measured at 340 nm. The glycogen content was calculated based on the glucose standard curve and the difference in the absorbance between digested and untreated samples. The protocol was adapted from Tennessen et al. [10].

### 2.4. Western Blotting

Previously isolated liver mitochondria were used for analysis, as well as liver tissue homogenised in RIPA lysis buffer (20188, Millipore, Billerica, MA, USA), supplemented with protease and phosphate inhibitors (P2714, Sigma-Aldrich; 524636, Millipore), after which the samples were centrifuged. Equivalent amounts (20 µg) of liver mitochondria/tissue were denatured in one volume of sample loading buffer and then separated on 12% gels by SDS-PAGE electrophoresis and transferred to PVDF membranes. Membranes were blocked with 5% non-fat dry milk or 5% BSA (in Tris-buffered saline, 0.1% Tween20) and incubated with the following primary antibodies: anti-Bax (Cell Signaling, Danvers, MA, USA #2772), anti-Bcl2 (Cell Signaling, #2870), anti-Beclin1 (Cell Signaling, #3495), anti-carbohydrate response element binding protein (ChREBP) (Santa Cruz, Dallas, TX, USA, sc-515922), anti-Cytochrome C (BD Biosciences, 556433), anti-dynamin related protein 1 (DRP1) (Cell Signaling, #8570), anti-estrogen receptor alpha (ERα) (Santa Cruz, sc-787), anti-microtubule-associated protein-1 light chain 3 (LC3) (MBL, Woburn, MA, USA, PD014), anti-mitofusin 1 (MFN1) (Santa Cruz, sc-50440), anti-mitofusin 2 (MFN2) (Santa Cruz, sc-50331), anti-optic atrophy 1 (OPA1) (Abcam, ab119685), anti-Parkin (Cell Signaling, #4211), anti-phosphoenolpyruvate carboxykinase, cytosolic (PEPCK-C) (Santa Cruz, sc-377027), anti-peroxisome proliferator-activated receptor gamma co-activator-1alpha (PGC-1α) (Abcam, Cambridge, UK, ab54481), anti-PTEN Induced Kinase 1 (PINK1) (Abcam, ab23707), anti-sterol regulator element binding protein 1 (SREBP-1) (Abcam, ab3259), anti-mitochondrial transcription factor A (TFAM) (Santa Cruz, sc-23588), anti-translocase of outer membrane 20 (TOM20) (Santa Cruz, sc-11415). Following this step, membranes were incubated with secondary antibodies: horseradish peroxidase-conjugated anti-goat, anti-mouse, or anti-rabbit (Santa Cruz, sc-2354, sc-2005, sc-2357, respectively). The dilutions of primary and secondary antibodies were given in Supplementary Table S1. To visualise the protein bands, membranes were incubated with Clarity Western ECL Substrate (Bio-Rad, Hercules, CA, USA, #1705061), and the signal was acquired by using ChemiDoc-XRS Image System (Bio-Rad). Eventual differences in protein loading and transfer were normalised by using Ponceau S staining [11]. The final data were expressed as the percentage variation of the control values (%P-C-S and %C-S for the mothers and offspring, respectively).

### 2.5. Real-Time PCR analysis

For the quantitative assessment of mRNA expression, total RNA was extracted from 30 mg of liver tissue using PureLinkTM RNA MiniKit (12183018A, ThermoFisher Scientific, Waltham, MA, USA) and then converted to cDNA using Xpert cDNA Synthesis MasterMix (#GK81, GRISP). Real-time PCR was performed using the fluorescent dye, PowerUp Sybr Green MasterMix (A25742, ThermoFisher Scientific), on a StepOnePlus thermocycler (Applied Biosystems, Waltham, MA, USA). The relative expression of target genes was normalised to that of 18S rRNA. The primers used are given in Table 1. For the miRNA analysis, small RNA was extracted from 20 mg of liver tissue using mirVana miRNA Isolation Kit (AM1560, Invitrogen, Waltham, MA, USA) and converted into cDNA using the TaqMan Advanced miRNA Assay (A25576, Applied Biosystems). The relative expressions of miR-122-5p, miR-34a-5p, miR-130b-3p, and miR-494-3p were determined using the TaqMan Fast Advanced MasterMix (Applied Biosystem). The primers used were assay ID rno480899_mir; rno481304_mir; rno481304_mir; rno481191_mir. Relative mRNA and miRNA expressions were reported as fold values of the control group (%P-C-S and %C-S for the mothers and offspring, respectively).

### 2.6. Quantification of Mitochondrial DNA Copy Number

To quantify the mtDNA copy number, total DNA was isolated from 30 mg of rats’ livers using GRS Genomic DNA Kit BroadRange (#GK06.0100, GRISP). Primers for mitochondrially encoded 16S RNA (Rnr2) were used for the detection of mtDNA, whereas for the detection of nDNA, primers for glyceraldehyde 3-phosphate dehydrogenase (GAPDH) were used. The specific primers are given in Table 1 [12]. Quantitative real-time PCR was conducted using PowerUp SYBR^®^ Green MasterMix (A25742, ThermoFisher Scientific) on a StepOnePlus thermocycler (Applied Biosystems). Mitochondrial and nuclear products were performed separately under the following conditions: 95 °C for 3 min, 40 cycles of 95 °C for 15 s and 60 °C for 1 min. The results were presented as mtDNA/nDNA (ΔCt = Ct(nDNA)–Ct(mtDNA); relative mtDNA content = 2 × 2^ΔCt^).

### 2.7. Statistical Analysis

The results are expressed as the mean ± SEM (standard error of the mean). Statistical analysis was performed in GraphPad Prism 9.0 software (GraphPad software, San Diego, CA, USA). Data were analysed using the one-way ANOVA test followed by the Sidak post hoc test, with the significance level set at 5%. The follow-up statistics were performed with the program RStudio Team (Integrated Development for R. RStudio, Boston, MA, USA).

## 3. Results

### 3.1. Regulators of Glucose/Lipid Metabolism

**Figure 1:**
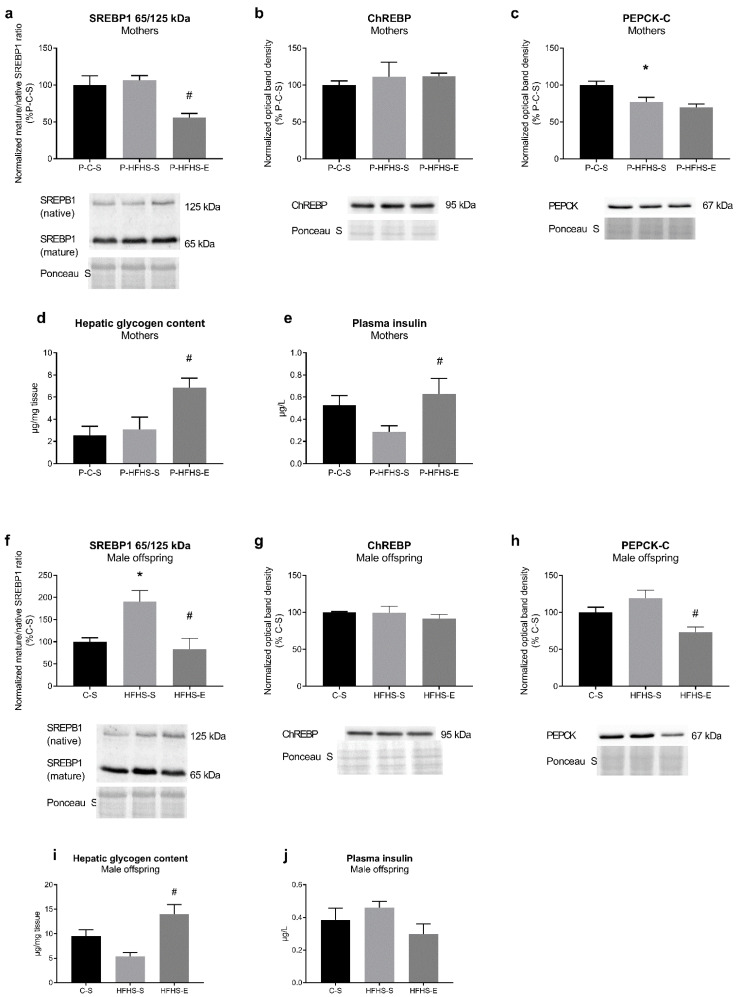
Effects of maternal HFHS and GE on regulators of liver glucose and lipid metabolism in mothers: (**a**) ratio of mature (65 kDa) and native (125 kDa) SREBP-1 form, (**b**) relative content of ChREBP, (**c**) relative content of PEPCK, (**d**) hepatic glycogen content, (**e**) plasma insulin levels; and in offspring: (**f**) ratio of mature and native SREBP-1 form, (**g**) relative content of ChREBP, (**h**) relative content of PEPCK, (**i**) hepatic glycogen content, (**j**) plasma insulin levels. GE—gestational exercise; C—mothers fed with control diet; HFHS—mothers fed with high-fat high-sucrose diet; S—sedentary mothers; E—exercised mothers. * vs. P-C-S (*p* < 0.05) or vs. C-S (*p* < 0.05) in mothers or offspring, respectively. # vs. P-HFHS-S (*p* < 0.05) or vs. HFHS-S (*p* < 0.05) in mothers or offspring, respectively.

Considering the role of transcriptional factors SREBP-1 and ChREBP in glucose and lipid metabolism, and PEPCK-C in gluconeogenesis, their protein amount was examined (Figure 1). In HFHS-fed mothers, no increase in mature/native SREBP-1 ratio was observed, while GE induced its decrease (Figure 1a). In HFHS-E offspring, the same effect of GE was observed; however, exposure to maternal HFHS diet increased SREBP-1 ratio in the offspring liver (Figure 1f). Although the HFHS diet in mothers lowered the PEPCK content (Figure 1c), a reduced PEPCK content in the offspring was only observed when mothers were exercised (Figure 1h). GE had the same stimulatory effect on plasma insulin in mothers (Figure 1e) and hepatic glycogen accumulation in both generations (Figure 1d,i).

### 3.2. Mitochondrial Biogenesis End Points

**Figure 2:**
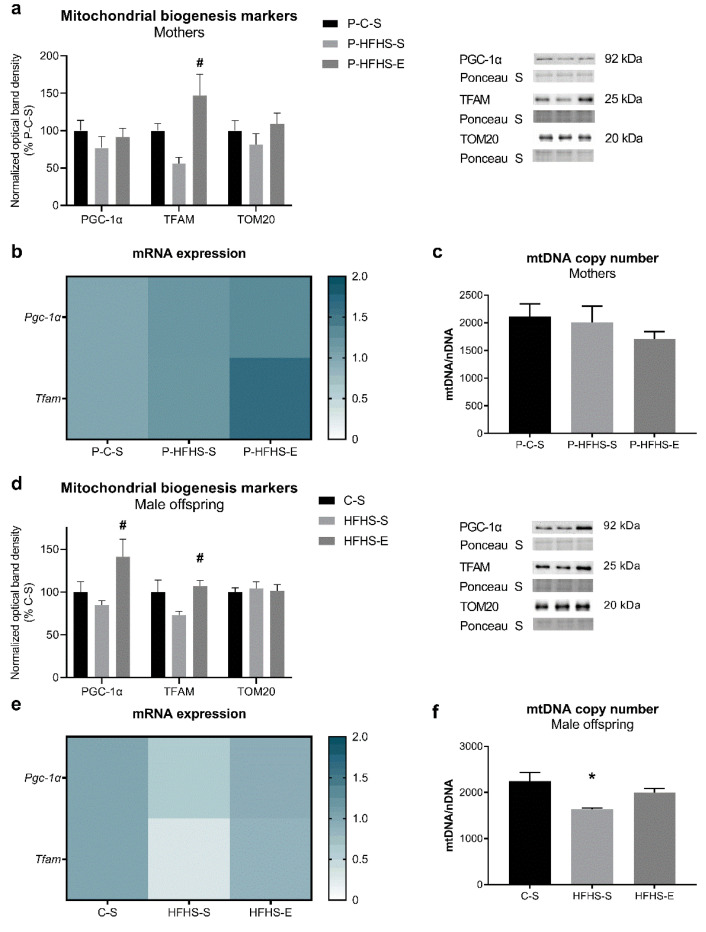

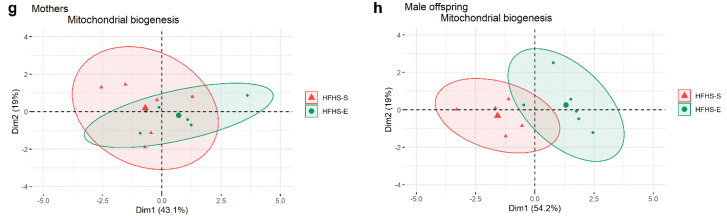
Effects of maternal HFHS and GE on different markers of liver mitochondrial biogenesis in mothers: (**a**) relative content of PGC-1α, TFAM, and TOM20, (**b**) heat map showing the relative expression levels for *Pgc*-*1α* and *Tfam* transcripts, (**c**) mitochondrial DNA copy number, (**g**) principal component analysis of hepatic mitochondrial biogenesis markers; and in offspring: (**d**) relative content of PGC-1α, TFAM, and TOM20, (**e**) heat map showing the relative mRNA expression levels of *Pgc-1α* and *Tfam*, (**f**) mitochondrial DNA copy number, (**h**) principal component analysis of hepatic mitochondrial biogenesis markers. GE—gestational exercise; C—mothers fed with control diet; HFHS—mothers fed with high-fat high-sucrose diet; S—sedentary mothers; E—exercised mothers. * vs. P-C-S (*p* < 0.05) or vs. C-S (*p* < 0.05) in mothers or offspring, respectively. # vs. P-HFHS-S (*p* < 0.05) or vs. HFHS-S (*p* < 0.05) in mothers or offspring, respectively.

The protein expressions of key regulators’ mitochondrial biogenesis (PGC-1α and TFAM) were assessed. Statistical differences were found between P-HFHS-S and P-HFHS-E regarding TFAM protein content in both mothers and offspring and PGC-1α only in offspring (Figure 2a,d). These effects of GE were also observed in the transcripts (Figure 2b,e). Furthermore, *Tfam* mRNA levels were remarkably reduced in HFHS-S offspring (*p* ≤ 0.01), which was reversed by GE (*p* ≤ 0.05) (Figure 2e). In contrast to the maternal, offspring mtDNA copy number was similarly altered by maternal diet/GE (Figure 2f). Yet, maternal diet/GE did not affect TOM20 content (Figure 2a,d). To further identify diet and exercise effects, principal component analysis (PCA), performed by using markers of mitochondrial biogenesis, showed a clear difference based on the maternal physical activity levels in the offspring of HFHS-fed mothers (Figure 2h), but not in mothers (Figure 2g), which could be a consequence of post-partum exercise cessation.

### 3.3. Mitochondrial Dynamics

**Figure 3:**
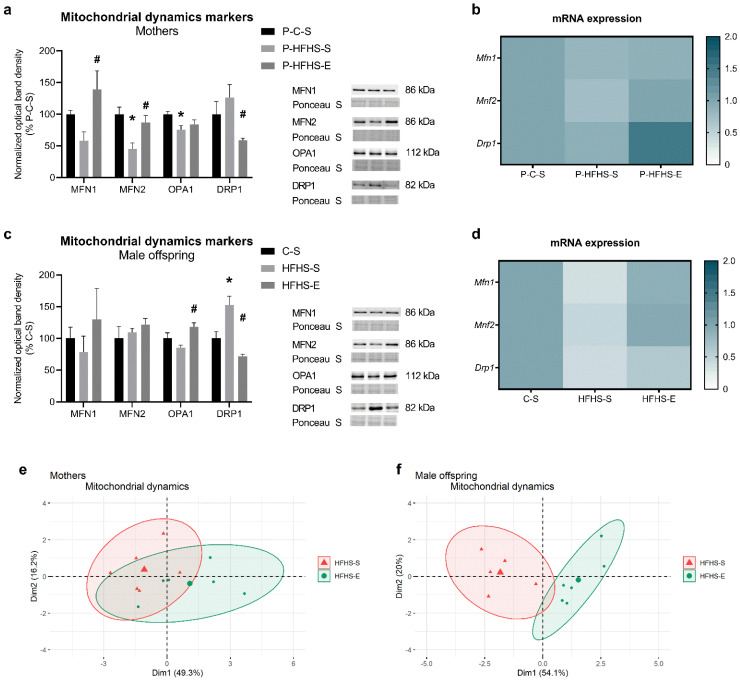
Effects of maternal HFHS and GE on different markers of liver mitochondrial dynamics in mothers: (**a**) relative mitochondrial content of MFN1, MFN2, OPA1, and DRP1, (**b**) heat map showing the relative transcripts for *Mfn1*, *Mfn2*, and *Drp1*, (**c**) principal component analysis of hepatic mitochondrial dynamics markers; and in the offspring: (**d**) relative mitochondrial content of MFN1, MFN2, OPA1, and DRP1, (**e**) heat map showing the relative hepatic transcripts for *Mfn1*, *Mfn2*, and *Drp1*, (**f**) principal component analysis of hepatic mitochondrial dynamics markers. GE—gestational exercise; C—mothers fed with control diet; HFHS—mothers fed with high-fat high-sucrose diet; S—sedentary mothers; E—exercised mothers. * vs. P-C-S (*p* < 0.05) or vs. C-S (*p* < 0.05) in mothers or offspring, respectively. # vs. P-HFHS-S (*p* < 0.05) or vs. HFHS-S (*p* < 0.05) in mothers or offspring, respectively.

The liver mitochondrial content of proteins involved in mitochondrial fusion (MFN1, MFN2, OPA1) and fission (DRP1) was analysed. In dams, HFHS feeding induced a reduction in the mitochondrial fusion proteins’ content, whereas GE reversed this effect in MFN1 and MFN2 contents but not in OPA1. DRP1 protein expression was increased 26% by HFHS diet in sedentary dams, whereas GE significantly reduced its content (Figure 3a). Furthermore, *Mfn1*, *Mfn2*, and *Drp1* transcripts tended to decrease due to HFHS diet and to increase in the exercising animals, although without a significant statistical difference (Figure 3b). Regarding the male offspring, most of the mitochondrial fusion proteins’ content was not significantly affected by maternal diet/GE; yet, GE slightly mitigated the decrease in OPA1 content imposed by the maternal HFHS diet. Similarly to their mothers, HFHS-S offspring had increased DRP1 protein expression, inhibited by GE, despite maternal HFHS diet (Figure 3c). Intrauterine exposure to maternal HFHS diet resulted in decreased transcripts for *Mfn1* (*p* ≤ 0.05), *Mfn2* (*p* = 0.06), and *Drp1* (*p* ≤ 0.01) in male offspring, which was prevented by GE (Figure 3d). As in mitochondrial biogenesis PCA (Figure 2g,h), the same effects of GE were observed regarding the mitochondrial dynamics’ markers (Figure 3e,f), undoubtingly showing the strong impact of GE despite altered intrauterine environment due to maternal HFHS diet.

### 3.4. Estrogen Receptor-α Protein Expression

**Figure 4:**
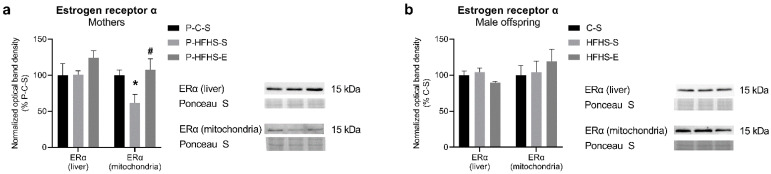
Effects of maternal HFHS and GE on the protein expression of ERα in the liver tissue and mitochondria of (**a**) maternal generation and (**b**) offspring generation. GE—gestational exercise; C—mothers fed with control diet; HFHS—mothers fed with high-fat high-sucrose diet; S—sedentary mothers; E—exercised mothers. * vs. P-C-S (*p* < 0.05) or vs. C-S (*p* < 0.05) in mothers or offspring, respectively. # vs. P-HFHS-S (*p* < 0.05) or vs. HFHS-S (*p* < 0.05) in mothers or offspring, respectively.

Since ER signalling has been associated with regulating metabolic function and is necessary for exercise-related protection against liver diseases [13], we determined the protein levels of ERα in liver tissue, as well as in liver mitochondria, where it can regulate mitochondrial biogenesis [14]. HFHS feeding in mothers reduced the mitochondrial ERα content, which was prevented by GE; yet, this was not observed at the liver tissue level (Figure 4a). In male offspring, no alterations were observed in ERα protein expression (Figure 4b).

### 3.5. Auto(mito)phagy Signalling

**Figure 5:**
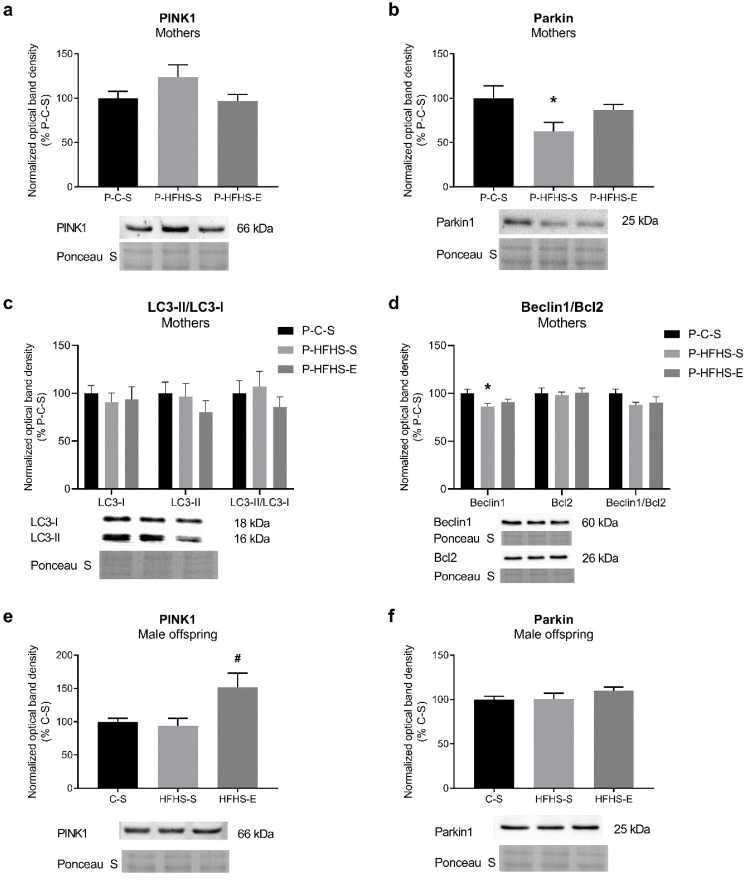

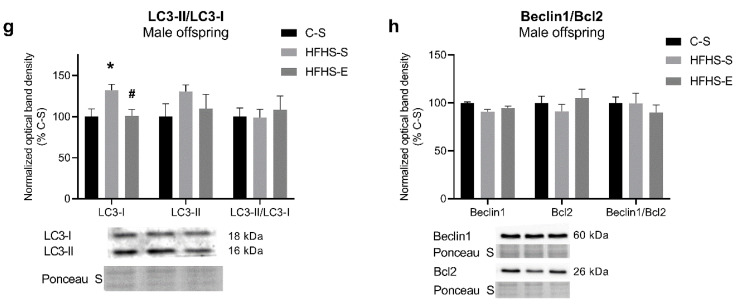
Effects of maternal HFHS and GE on the protein expression of different markers of auto(mito)phagy signalling in liver and liver mitochondria in mothers: (**a**) relative content of PINK1, (**b**) relative content of Parkin, (**c**) relative content of LC3-I and LC3-II and their ratio, (**d**) relative content of Beclin1 and Bcl2 and their ratio; and in offspring: (**e**) relative content of PINK1, (**f**) relative content of Parkin, (**g**) relative content of LC3-I and LC3-II and their ratio, (**h**) relative content of Beclin1 and Bcl2 and their ratio. GE—gestational exercise; C—mothers fed with control diet; HFHS—mothers fed with high-fat high-sucrose diet; S—sedentary mothers; E—exercised mothers. * vs. P-C-S (*p* < 0.05) or vs. C-S (*p* < 0.05) in mothers or offspring, respectively. # vs. P-HFHS-S (*p* < 0.05) or vs. HFHS-S (*p* < 0.05) in mothers or offspring, respectively.

Autophagy and mitophagy being important mechanisms for cell quality control processes, coupled with mitochondrial biogenesis and dynamics, we assessed the content of proteins PINK1 and Parkin, and LC3-II, pro-autophagic Beclin1, and anti-autophagic and anti-apoptotic Bcl2. The hepatic levels of these proteins were not significantly affected by maternal diet/GE in mothers or offspring. HFHS feeding reduced Parkin content in dams, while GE increased it by 38% (Figure 5b). The same effect was observed in the pro-autophagic Beclin1 content (Figure 5d). The HFHS-E offspring showed increased PINK1 expression (Figure 5e). However, the HFHS-S offspring had a higher LC3-I protein content and the same trend in LC3-II, which was rescued by GE (Figure 5g). The Beclin1/Bcl2 ratio was not altered in any of the groups studied.

### 3.6. Apoptotic Signalling

**Figure 6:**
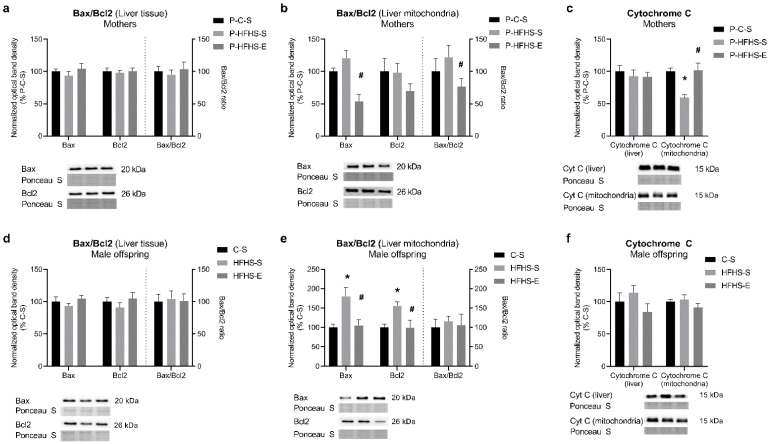
Effects of maternal HFHS and GE on the protein expression of different markers of apoptotic signalling in mothers: (**a**) relative content of liver Bax and Bcl2 and their ratio, (**b**) relative content of liver mitochondrial Bax and Bcl2 and their ratio, (**c**) relative content of liver and liver mitochondrial cytochrome c; and in offspring: (**d**) relative content of liver Bax and Bcl2 and their ratio, (**e**) relative content of liver mitochondrial Bax and Bcl2 and their ratio, (**f**) relative content of liver and liver mitochondrial cytochrome c. GE—gestational exercise; C—mothers fed with control diet; HFHS—mothers fed with high-fat high-sucrose diet; S—sedentary mothers; E—exercised mothers. * vs. P-C-S (*p* < 0.05) or vs. C-S (*p* < 0.05) in mothers or offspring, respectively. # vs. P-HFHS-S (*p* < 0.05) or vs. HFHS-S (*p* < 0.05) in mothers or offspring, respectively.

Analysis of the content of some pro-apoptotic proteins at the tissue level showed no alterations in any generation (Figure 6). Nevertheless, the same proteins were remarkably modulated at the mitochondrial level, particularly in the maternal generation, suggesting a delicate adjustment of these mechanisms to diet and physical activity levels. Mitochondrial pro-apoptotic Bax and its ratio over Bcl2 increased by 38% in HFHS-fed mothers, which was prevented by GE (Figure 6b). Opposite effects were observed in mitochondrial cytochrome c content (Figure 6c), suggesting that cytochrome c release occurred in the P-HFHS-S group and was prevented even with a short exercise period. Maternal HFHS increased offspring Bax and Bcl2 content, which was reversed by GE (Figure 6e). However, it seems that maternal diet and exercise were not sufficiently strong factors to modulate some of the proteins involved in apoptotic pathways in the offspring.

### 3.7. Expression of miR-122, miR-34a, miR-130b, and miR-494 in the Liver

**Figure 7:**
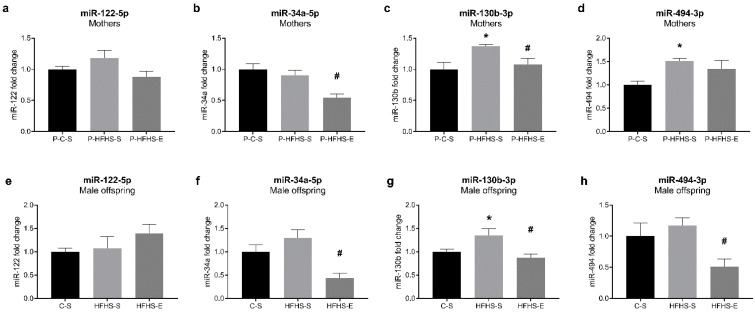
Effects of maternal HFHS and GE on the micro-RNA expression in maternal livers: (**a**) relative expression of miR-122, (**b**) relative expression of miR-34a, (**c**) relative expression of miR-130b, (**d**) relative expression of miR-494; and in offspring: (**e**) relative expression of miR-122, (**f**) relative expression of miR-34a, (**g**) relative expression of miR-130b, (**h**) relative expression of miR-494. GE—gestational exercise; C—mothers fed with control diet; HFHS—mothers fed with high-fat high-sucrose diet; S—sedentary mothers; E—exercised mothers. * vs. P-C-S (*p* < 0.05) or vs. C-S (*p* < 0.05) in mothers or offspring, respectively. # vs. P-HFHS-S (*p* < 0.05) or vs. HFHS-S (*p* < 0.05) in mothers or offspring, respectively.

As epigenetic regulation could help explain the intergenerational effect of maternal diet/GE observed in this study, we examined the microRNAs that regulate hepatic lipid metabolism and/or mitochondrial biogenesis. Even though miR-122 is one of the most abundant hepatic microRNAs involved in lipid metabolism [7], no alterations were observed in any generation (Figure 7a,e). However, miR-34a, known to inhibit PGC-1α activation [15], was reduced by GE in both mothers and offspring (Figure 7b,f), which concurs with PGC-1α regulation (Figure 2). With similar roles, miR-130b expression was stimulated by HFHS diet but prevented by GE (Figure 7c,g) following regulation of PGC-1α. Interestingly, miR-494, targeting TFAM [16], was increased by HFHS diet in mothers and only slightly in the offspring, whereas GE decreased its levels only in the offspring generation (Figure 7d,h).

## 4. Discussion

Maternal nutrition alterations during pregnancy and/or GDM may induce metabolic alterations during early embryonic and foetal life and trigger chronic metabolic disorders in offspring [1]. However, GE can prevent these adverse effects on metabolic health and reduce the risk of NAFLD in high-fat diet (HFD)-fed offspring [17]. Recently, our group suggested that GE mitigated liver lipid accumulation and mitochondrial dysfunction in male offspring of GDM mothers and prevented some HFHS deleterious consequences in mothers [9]. Therefore, we aimed to clarify the putative mechanisms behind these GE-modulated metabolic and mitochondrial manifestations, focusing on mitochondrial biogenesis and dynamics and quality control signalling in mothers and male offspring.

Considering the previously observed effects of maternal diet/GE on hepatic triglyceride accumulation [9], we analysed the SREBP-1 and ChREBP contents as vital transcription factors involved in de novo lipogenesis. ChREBP was not affected in our study, while the lower mature/native SREBP-1 ratio in exercising mothers and their male offspring supports the decreased hepatic triglyceride accumulation observed in our previous study [9], as SREBP-1 knock-down lowered hepatic triglycerides content [18]. SREBP-1 is activated by insulin and ChREBP by glucose, independently of insulin [19], and since increased SREBP-1 is related to IR [20], it could be speculated that GDM characteristic IR was a more important factor in affecting the male offspring lipid metabolism. Contrary to expectations, miR-122, as a liver-specific microRNA targeting SREBP-1 [7], was not affected by maternal diet/GE. Reduced hepatic miR-122 was reported in the offspring of HFD-fed mice [21] and male foetuses of GDM mothers [22]. In diet-induced diabetic and NAFLD rats, exercise improved NAFLD phenotype by inducing hepatic miR-122 and downregulating its target genes [7]. Whether offspring miR-122 content was not susceptible to specific intrauterine conditions in our study or there is a reversal effect of the offspring C diet post-weaning consumption deserves further studies. In a healthy liver, miR-122 and miR-34a cooperatively regulate and balance cell survival and proliferation [23], with miR-34a being upregulated in the liver of HFD-fed mice [24] and steatohepatitis patients [25]. Despite non-statistically significant miR-34a increase in the offspring of GDM mothers, GE remarkably inhibited its expression in both generations. Similarly, swimming suppressed brain miR-34a expression, thus rescuing impaired autophagy and mitochondrial dynamics in brain aging disorders [26], whereas voluntary exercise in HFD rats reduced testicular miR-34a and related apoptosis [27]. Still, the effect of exercise on miR-34a levels is not fully understood, particularly at the intergenerational level.

Additionally, reduced SREBP-1 gene expression and increased hepatic glycogen accumulation in exercised HFHS mothers and their offspring corroborate the finding that SREBP-1 knock-down upregulates genes associated with hepatic glycogenesis and gluconeogenesis [18]. Hepatic gluconeogenesis is found to increase in NAFLD and diabetes [28,29], which is prevented by acute exercise together with an increased hepatic glycogen content [30] or by chronic exercise through improvement of insulin sensitivity [28], and agrees with our data. Gluconeogenesis tends to increase during pregnancy, regardless of GDM [30]. Yet, as the reliance on gluconeogenesis is lower when glucose is directly available from external resources [29], low PEPCK-C levels after 18-week-long sucrose-rich feeding in our study are not surprising, with 3 weeks of exercise not being enough to counteract this effect. Nevertheless, miR-34a changes can explain, at least partially, offspring hepatic glycogen and PEPCK regulation. Hepatic miR-34a overexpression was associated with increased *Pepck* transcripts [31]. Additionally, miR-34a downregulation restored fibroblast growth factor-19 [31] and -21 signalling [32], which further restored glycogen loss in diabetic animals [33] and eventually supressed SREBP-1 [34] and PEPCK [35], respectively. Additionally, enhanced PGC-1α was detected in miR-34a knock-out mice [15], which corroborates our data. In fact, lipogenic SREBP-1c can interfere and inhibit PGC-1α recruitment, thus suppressing hepatic PEPCK [36]. Therefore, SREBP-1 alterations, at least partly and indirectly regulated by miR-34a in the offspring of exercising mothers, could explain previously observed changes in hepatic lipid accumulation of male offspring [9].

Indeed, excessive lipid accumulation is associated with impaired activity of PGC-1α and thus decreased mitochondrial biogenesis, consequently leading to decreased β-oxidation and respiratory function [37], which we also observed in the liver of male offspring of sedentary HFHS mothers [9]. Impaired mitochondrial biogenesis, observed as reduced PGC-1α and TFAM and mtDNA content, was found in NAFLD mice [37] and diabetic patients [38], which might be related to their reduced exercise capacity [39]. It is also impaired in diabetic women’s placenta, with PGC-1α, TFAM, and mtDNA content reduced in the placenta of male but not female offspring [40], posing a possible risk of metabolic disturbances in adulthood. Indeed, our data agree with these findings. Although in GDM mothers, GE increased the PGC-1α content to some extent, prolonged HFHS consumption and exercise cessation were the probable reason for not reverting it to the control level. Still, its downstream target TFAM suggested potentially impaired mitochondrial biogenesis in GDM mothers, which was reversed by exercise. Similarly, in the offspring, GE promoted PGC-1α and TFAM expression and the generation of new mitochondria, as supported by increased mtDNA copy number. Additionally, liver PGC-1α upregulation can reduce triglyceride accumulation and prevent impaired mitochondrial respiration [41]—effects we reported previously [9] and which could be, at least partly, attributed to PGC-1α overexpression. Mitochondrial biogenesis can be further regulated by the translocation of ERα into mitochondria. A prolonged over-stimulation or deficiency of ER-mediated mitochondrial biogenesis can lead to over-accumulation or deficiency of ATP and ROS, and generally, mitochondrial dysfunction [14]. Interestingly, maternal diet/GE rather affected ERα content in liver mitochondria than on the tissue level, suggesting the importance of ERα-related mitochondrial biogenesis during pregnancy. Additionally, ERα signalling was involved in exercise-related beneficial effects against NAFLD, with some of them being limited with ERα loss [13]. Still, this association could not be observed in the offspring, probably due to young age, before their estrogen synthesis and regulation began [42].

MiR-130b overexpression has been associated with decline in PGC-1α and TFAM content in human placenta in diabetic conditions [43]. Additionally, maternal GDM stimulated miR-130b expression and SREBP-1 ratio in both generations while downregulating PGC-1α and TFAM [44]. Although some exercise modalities can decrease circulating miR-130b [45], the effects of exercise on liver miR-130b expression are unknown. To our knowledge, this is the first study showing that GE-related reduction in hepatic miR-130b levels is associated with alterations in PGC-1α in mothers and male offspring. Exercise can downregulate skeletal muscle miR-494 expression as well and thus upregulate TFAM expression [16], which was also evident in the offspring of exercised HFHS mothers. Although peripheral blood miR-494 is reportedly downregulated in GDM women [46], GDM mothers had upregulated hepatic miR-494, regardless of exercise, suggesting that miR-494 effects might be tissue specific and that exercise cessation influenced its expression.

Maternal nutrient oversupply can also affect mitochondrial dynamics processes, leading to eventual metabolic compromise [47]. Hyperglycaemia and hyperlipidaemia can shift mitochondria towards fission over fusion, favouring mitochondrial fragmentation and decreasing mitochondrial generation ATP capacity [48]. A similar effect of GDM-related hyperglycaemia was noticed in liver mitochondria of GDM dams and their offspring, agreeing with observed PGC-1α content. The PGC-1α pathway regulates MFN2 [49], whose deficiency has been associated with IR and diabetes [38,48], followed by PGC-1α deficiency as well [38]. Reduced MFN2 levels and related mitochondrial damage and apoptosis were observed in the placenta of early miscarriages [50], suggesting that precise regulation of mitochondrial dynamics is necessary for some developmental processes [51]. Our data suggest that mitochondria may favour fission to remove dysfunctional mitochondria and preserve bioenergetics in GDM dams and their offspring. Regardless of the different regulation of mitochondrial fusion in the two generations, GE improved mitochondrial biogenesis and dynamics, thus stimulating an adaptive response for mitochondrial renewal. In high-energy-demanding conditions, such as exercise, mitochondria tend to fuse and extend their network, providing benefits to cellular homeostasis [52]. Interestingly, among fusion proteins, the offspring of exercised mothers had only significantly higher OPA1 levels, partly explained by the improved mitochondrial oxidative phosphorylation (OXPHOS) capacity observed in this offspring group [9]. Under high OXPHOS levels, OPA1 activity at the inner mitochondrial membranes is stimulated, whereas mitochondrial outer membrane fusion seems to be insensitive to OXPHOS regulation [53]. On the other hand, OPA1 might be important for regulating OXPHOS, as OPA1-depleted cells expressed impairments in OXPHOS [54]. GE also prevented increasing mitochondrial fission protein content imposed by maternal GDM in both generations. As the activation of DRP1-mediated fission can respond to the presence of damaged mitochondria, exercise might be an important factor shaping offspring mitochondria in the very early stage of foetal development.

In sedentary dams, HFHS diet upregulated DRP1 content but downregulated Parkin expression without affecting PINK1, which regulates mitophagy through Parkin and DRP1 activation in response to mitochondrial damage. Yet, PINK1 activation is not mandatory for mitophagy, since DRP1 could eventually successfully contribute to removing damaged mitochondria [55]. Decreased Parkin levels, and thus decreased stimulation of mitophagy, may suggest an impaired removal of damaged mitochondria enforced by HFHS and sedentary lifestyle in dams. In HFD-induced steatohepatitis, Parkin was also reduced in sedentary animals, whereas exercise restored mitophagy signalling [3]. We did not observe such effect of GE in dams, which could be ascribed to the post-partum exercise cessation, strengthening the need for regular physical activity. Still, GE stimulated offspring PINK1 protein expression. PINK1 can ensure the rapid segregation of damaged mitochondria and faster mitophagy, without necessary over-activation of DRP1 [55]. Moreover, PINK1 accumulation can reduce mtDNA replication to limit the damaged mtDNA transmission [56], which agrees with our results, suggesting that GE is an important factor during intrauterine development, which maintains a healthy mitochondrial pool in male offspring. However, we did not observe any alteration in hepatic apoptotic signalling. Likewise, the Bax expression was not affected in the placenta of diabetic women [50]. Since regulation of mitochondrial fusion and fission is associated upstream with apoptosis [50,57], it is not surprising that alterations in apoptotic signalling were only observed at the liver mitochondrial but not tissue level in our study. Bax translocation and accumulation in mitochondria under apoptotic stimuli can be an early event contributing to the cytochrome c release from mitochondria and initiation of the intrinsic apoptotic signalling [58]. Accordingly, the cytochrome c content in mothers was greatly reduced in liver mitochondria but not at the tissue level, which GE successfully prevented. Although the mitochondrial isolation protocol may cause loss of some cytochrome c, which may be more extensive in some groups than in others, this result suggests that, physiologically, an increased cytochrome c release occurs in HFHS-fed mothers Still, Bax accumulation might not be the only cause of the lower mitochondrial cytochrome c content, as fission-related DRP1 is also involved in apoptotic signalling through cytochrome c [57]. Mitochondrial DRP1 levels were indeed increased in sedentary HFHS dams, which, combined with OPA1 depletion [59], might have facilitated a potential cytochrome c release and contributed to a slight pro-apoptotic image in GDM dams. Yet, such changes were not observed in the male offspring, and despite eventual regulation of Bax and Bcl2 protein expression, maternal diet/GE did not modulate apoptotic signalling, at least in this age, sex, and tissue.

## 5. Conclusions

Generally, GE alleviated hepatic metabolic disturbances in GDM mothers despite prolonged HFHS feeding and exercise cessation. The impact of GE was more obvious and influential in the offspring. GE prevented the over-excessive placental transfer of maternal food-derived nutrients to the foetal liver, thus reducing the nutritional pressure on offspring liver mitochondria and enabling normal development of liver structure and function and mitochondrial biogenesis, dynamics, and quality control signalling. Our results revealed that changes caused by diet and GE are more evident in liver mitochondria rather than in the whole liver tissue, either directly or via intrauterine environment. In the absence of interventions, these mitochondrial alterations and the prolonged harmful lifestyle may lead to a more exacerbated metabolic phenotype.

## Figures and Tables

**Table 1:** PCR primer sequences.

Gene	Forward Primer Sequence (5′-3′)	Reverse Primer Sequence (5′-3′)
Pgc-1α	AAAAGCTTGACTGGCGTCAT	TCAGGAAGATCTGGGCAAAG
Tfam	GCTAAACACCCAGATGCAAAA	CGAGGTCTTTTTGGTTTTCC
Mfn1	TGGTCACACAACCAACTGCT	GGGCCAAAATACGTGCACAA
Mfn2	GTGACGTGTTGGGTGTGAT	GGACATCTCGTTTCTAGCTGGT
Drp1	CCAGGAATGACCAAGGTCCC	CCTCGTCCATCAGGTCCAAC
18S rRNA	CATTCGAACGTCTGCCCTAT	GTTTCTCAGGCTCCCTCTCC
GAPDH (nuclear DNA) [12]	GGAAAGACAGGTGTTTTGCA	AGGTCAGAGTGAGCAGGACA
Rnr2 (mitochondrial DNA) [12]	AGCTATTAATGGTTCGTTTGT	AGGAGGCTCCATTTCTCTTGT

## Data Availability

The data presented in this study are available on request from the corresponding author. The data are not publicly available due to privacy.

## Supplementary Materials

The following supporting information can be downloaded at: https://www.mdpi.com/article/10.3390/ijerph20021388/s1, Table S1: List of antibodies and dilutions used in the western blotting.
