# Supplementary material for: Gestational Exercise Antagonises the Impact of Maternal High-Fat High-Sucrose Diet on Liver Mitochondrial Alterations and Quality Control Signalling in Male Offspring
Source: Int J Environ Res Public Health. 2023 Jan 12;20(2):1388. doi: 10.3390/ijerph20021388 (PMC9858977; doi:10.3390/ijerph20021388)
Supplement: Supplementary file 1 [file ijerph-20-01388-s001.zip › ijerph-2111587-supplementary.pdf]

International Journal of
Environmental Research and Public Health

MDPI

Supplementary Table S1. List of antibodies and dilutions used in the western blotting.

Antibody	Manufacturer	Dilution
Bax	Cell Signaling, #2772	1:1000
Bcl2	Cell Signaling, #2870	1:1000
Beclin1	Cell Signaling, #3495	1:750
ChREBP	Santa Cruz, sc-515922	1:800
Cytochrome C	BD Biosciences, 556433	1:1000
DRP1	Cell Signaling, #8570	1:500
ERα	Santa Cruz, sc-787	1:500
LC3	MBL, PD014	1:750
MFN1	Santa Cruz, sc-50440	1:500
MFN2	Santa Cruz, sc-50331	1:500
OPA1	Abcam, ab119685	1:750
Parkin	Cell Signaling, #4211	1:500
PEPCK-C	Santa Cruz, sc-377027	1:500
PGC-1α	Abcam, ab54481	1:1000
PINK1	Abcam, ab23707	1:750
SREBP-1	Abcam, ab3259	1:1000
TFAM	Santa Cruz, sc-23588	1:500
TOM20	Santa Cruz, sc-11415	1:1000
horseradish peroxidase-conjugated anti-goat	Santa Cruz, sc-2354	1:8500
horseradish peroxidase-conjugated anti-mouse	Santa Cruz, sc-2005	1:10000
horseradish peroxidase-conjugated anti-rabbit	Santa Cruz, sc-2357	1:5000

ChREBP - carbohydrate response element binding protein.
DRP1 - dynamin related protein 1.
ERα - estrogen receptor alpha.
LC3 - microtubule-associated protein-1 light chain 3.
MFN1- mitofusin 1.
MFN2 - mitofusin 2.
OPA1 - optic atrophy 1.
PEPCK-C - phosphoenolpyruvate carboxykinase, cytosolic.
PGC-1α - peroxisome proliferator-activated receptor gamma co-activator-1alpha.
PINK1 - PTEN Induced Kinase 1.
SREBP-1 - sterol regulator element binding protein 1.
TFAM - mitochondrial transcription factor A.
TOM20 - translocase of outer membrane 20.
